# Safety and efficacy of bevacizumab biosimilar in recurrent/ progressive glioblastoma

**DOI:** 10.3332/ecancer.2021.1166

**Published:** 2021-01-13

**Authors:** Gunjesh Kumar, Hollis DSouza, Nandini Menon, Sujay Srinivas, Dilip Harindran Vallathol, Mounika Boppana, Annu Rajpurohit, Abhishek Mahajan, Amit Janu, Abhishek Chatterjee, Rahul Krishnatry, Tejpal Gupta, Rakesh Jalali, Vijay M Patil

**Affiliations:** 1 Department of Medical Oncology, Tata Memorial Hospital, Parel 400012 Mumbai, India; 2 Department of Radiodiagnosis, Tata Memorial Hospital, Parel 400012 Mumbai, India; 3 Department of Radiation Oncology, Tata Memorial Hospital, Parel 400012 Mumbai, India; † Co-first authorship

**Keywords:** bevacizumab, biosimilar, innovator, glioma

## Abstract

**Background:**

Multiple low-cost biosimilars of bevacizumab are now available but their clinical efficacy has never been compared against the original (innovator) molecule in glioblastoma. The aim of the current analysis is to compare the overall survival (OS) in recurrent/progressive glioblastoma patients between the biosimilar and innovator molecules.

**Materials and methods:**

Adult recurrent/progressive glioblastoma patients treated with bevacizumab from 1 July 2015 to 30 July 2019 were identified. These patients were either offered Bevacizumab innovator (Avastin, Roche) or biosimilar (BevaciRel: Reliance Life sciences or Bryxta: Zydus Oncosciences) depending upon the financial status and affordability of the patients. The primary endpoint of the study was OS, while progression-free survival (PFS) and adverse events were the secondary endpoints.

**Results:**

There were 82 patients, out of which 57 received innovator and 25 received biosimilar bevacizumab. At median follow-up of 26 months, the median PFS was 3.66 (95% confidence interval (CI) 2.08 to 5.25) and 3.3 months (95% CI 2.38 to 4.21) in innovator and biosimilar group, respectively (Log-rank test *p*-value = 0.072). The hazard ratio (HR) for progression was 0.61 (95% CI 0.35 to 1.05; *p*-value = 0.075). At the time of data cut-off, the median OS was 5.53 (95% CI, 5.07 to 5.99) versus 7.33 months (95% CI, 5.63 to 9.03) in innovator and biosimilar group, respectively (Log-rank test *p*-value = 0.51). The HR for death was 1.21 (95% CI, 0.67 to 2.17; *p*-value = 0.51). The adverse events and safety profiles were comparable between the two groups.

**Conclusion:**

In the recurrent/progressive glioblastoma patients, both innovator and biosimilar bevacizumab seem to have similar safety and clinical efficacy.

## Background

High grade gliomas are best treated with a multidisciplinary approach [1]. However, in spite of adequate treatment, most of the patients show recurrence. The median time to progression (TTP) for grade IV glioma/glioblastoma and grade 3 astrocytoma is 6.9 (95% CI 5.8–8.2 months) and 42.8 months (95% CI 28.6–60.6), respectively [2]. The decision on the type of treatment in cases of disease progression or recurrence depends upon the Eastern Cooperative Oncology Group-Performance Status (ECOG PS), TTP or relapse, site of recurrence, size of recurrence and previous treatment [3]. Re-surgery and re-irradiation arguably are considered the treatments of choice in such cases. However, very few patients qualify for these options and most of them end up receiving palliative systemic therapy including Bevacizumab which is one of the important drugs in the oncologist’s armamentarium.

Bevacizumab is a monoclonal antibody against vascular endothelial growth factor (VEGF) which has been implicated in the pathogenesis of progressive glioblastoma [4]. Bevacizumab alone or in combination with chemotherapy is used routinely in the clinical practice for different malignancies. It received an accelerated approval in 2009 for the treatment of progressive glioblastoma after the BRAIN trial [5]. Though it failed to improve overall survival (OS) against lomustine (CCNU) in the EORTC 26101 study, the improvement in progression free survival (PFS) was considered significant and the drug received final approval by the Food and Drug Association (FDA) in the year 2017 for progressive glioblastoma [6, 7].

In spite of the approval, Bevacizumab is not very popular among the clinicians in the low- and middle-income countries and its widespread utilisation still remains hampered owing to the high costs similar to any other monoclonal antibody. Bevacizumab (Avastin, Roche) lost its patent in 2016 and multiple biosimilars entered the Indian market the same year. These biosimilars were available at lower prices than the original (innovator) molecule (Avastin, Roche). They soon took over the market and are now widely prescribed. Whether these biosimilars have same efficacy as the original drug in the treatment of progressive glioblastoma remains an open question that still needs to be answered.

## Methods

### Selection of patients

The neuro-medical oncology unit has maintained a prospective database of all the patients undergoing chemotherapy since 1 July 2015. This database was used for the current analysis. Patients were selected from this database using the below mentioned criteria:

1. Adult patient aged ≥ 18 years
2. Relapsed or progressive glioblastoma
3. Treated with bevacizumab
4. Time period from 1 July 2015 to 30 November 2019

Patients satisfying all four criteria were selected and the data with regard to the age, gender, category (private or general), diabetes, ischemic heart disease (IHD) and smoking habit were extracted and entered in an excel sheet. Patients of both categories—general and private participated in the study. The general category patients were either not charged or minimally charged for the consultation and investigations, whereas private category ones were fully charged for the same.

### Treatment

All the patients were discussed in the joint neuro-oncology meeting. These patients were ineligible for re-surgery or re-irradiation and were offered bevacizumab either alone or in combination with cytotoxic therapy. The choice of single agent therapy or combination was based on the ECOG PS and presence of co-morbidities. Bevacizumab was administered every 2–3 weeks. The fisrt dose was given with adequate supportive medications over 90 minutes and the subsequent doses were administered in 30 minutes. The drug was continued till either disease progression or intolerable side effects. The drug was also stopped if the financials of the patient were inadequate.

### Data collection

The detailed baseline characteristics, previous treatment, histopathology, molecular features, bevacizumab start date, brand of bevacizumab; Avastin: Roche (innovator) or BevaciRel: Reliance Life sciences and Bryxta: Zydus Oncosciences (biosimilar), date of progression and date of death were noted in the excel sheet.

### Endpoints

The primary endpoint of the study was OS. It was defined as time in months from start of bevacizumab to death. For the patients who were still alive at the time of data censoring the OS was calculated up to the date of last follow-up. The secondary endpoints were PFS and adverse events. PFS was defined as time in months from the start of bevacizumab to progression or death. For patients who were alive and had not progressed at the time of data censoring, this time interval was calculated up to the date of last follow-up. The adverse events were graded according to the common terminology criteria for adverse events version 4.02.

### Statistical analysis

SPSS version 20 and R studio version 3.5.2 were used for the analysis. The time to event variables were estimated using the Kaplan–Meier method. The median with its 95% CI was calculated using the Brookmeyer and Crowley method. The estimates were compared between the original and biosimilar bevacizumab cohorts using the log rank test. The hazard ratio (HR) was calculated using Cox regression analysis. The assumption for proportionality was tested before forming the Cox regression analysis and they were met. The continuous variables were expressed in terms of median with 95% CI and compared between the two cohorts using the median test. The normal distribution of continuous variables was confirmed using the Shapiro–Wilk test. The ordinal and nominal variables were expressed in the terms of percentage with 95% CI and were compared between the two cohorts using the Fisher’s test.

## Results

### Baseline characteristics

The total enrolled patients were 82. Out of total, 57 received innovator (original) and 25 received biosimilar bevacizumab. 87.7% patients from innovator and 80% from biosimilar cohort belonged to the younger age group (18–59 years).

Both sets in the study had predominantly males constituting 77.2% and 80% of the total patients in the innovator and biosimilar groups, respectively. In the innovator group, 44 out of 57 (73.7%) were private patients, while 13 (26.3%) patients were from the general category whereas in the biosimilar group, 13 out of 25 (52%) belonged to the private and 12 (48%) belonged to the general category and the differences between them were statistically significant (Fisher’s exact test, *p*-value = 0.028). Further details of baseline characteristics are mentioned in the Table 1.

### Molecular characteristics

The molecular analysis showed no significant difference between the two groups in terms of isocitrate dehydrogenase (IDH) mutation (Fisher’s exact test, *p*-value = 0.143), while the differences were statistically significant for O6-methylguanine-DNA methyl-transferase (MGMT) methylation (Fisher’s exact test, *p*-value = 0.007), 1p19q deletion (Fisher’s exact test, *p*-value = 0.001) and telomerase reverse transcriptase (TERT) promoter mutation (Fisher’s exact test, *p*-value = 0.000) (Table 2).

### Outcome

**Figure 1.**
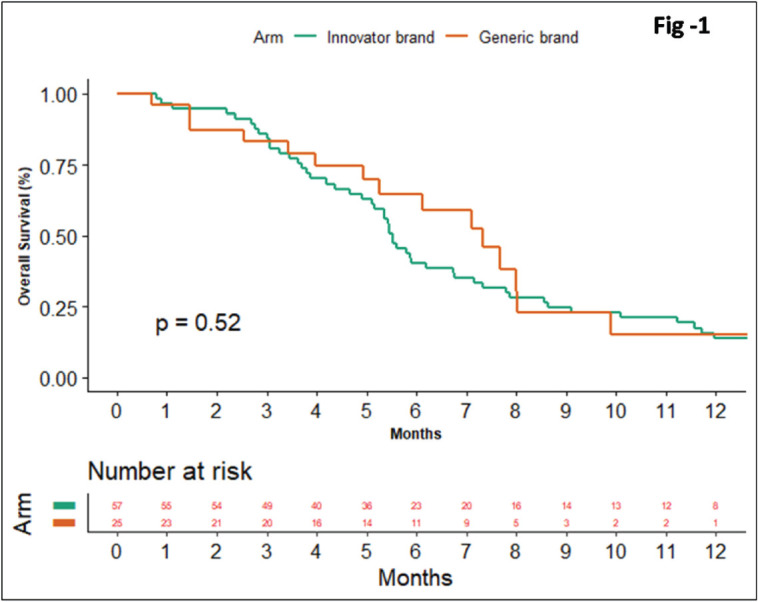
Comparison of OS between innovator and biosimilar bevacizumab.

At the median follow-up of 26 months, 76 patients had an event for progression. The median PFS was 3.66 (95% CI 2.08 to 5.25) and 3.3 months (95% CI 2.38 to 4.21) in the innovator and biosimilar group, respectively (Log-rank test *p*-value = 0.072). The HR for progression was 0.61 (95% CI 0.35 to 1.05; *p*-value = 0.075). At the time of data cut-off, there were 69 deaths. The median OS was 5.53 (95% CI, 5.07 to 5.99) versus 7.33 months (95% CI, 5.63 to 9.03) in innovator and biosimilar group, respectively (Log-rank test *p*-value = 0.51). The HR for death was 1.21 (95% CI, 0.67 to 2.17; *p*-value = 0.51) (Figure 1).

### Adverse events

A summary of adverse events is shown in Table 3. The observed adverse events were mostly anti-VEGF toxicities including hypertension, thrombosis, bleeding, proteinuria and dyslipidaemia, along with others such as febrile neutropenia, diarrhoea, myelosuppression, transaminitis, hypersensitivity and fatigue. These events were comparable between the two treatment groups. Also, there was no difference in grade 3 and grade 4 toxicities between the two groups (Fischer’s exact test, *p*-value = 0.373).

## Discussion

Glioblastoma Multiforme (GBM) is one of the deadliest tumours and accounts for 0.5% of all malignancies in India [8]. Due to its aggressive nature and short TTP, bevacizumab has an important place in the treatment of GBM especially in the recurrent setting. It is not the first time when biosimilar of an original molecule has been tried and documented in the literature. Filgrastim (2015) and Infliximab (April 2016) were the first biosimilars approved by the United States (US) FDA. Later, biosimilars for etanercept (August 2016) and adalimumab (September 2016) were also approved [9]. Trastuzumab and rituximab biosimilars have also been tried, found to be clinically efficient and hence have been approved by the FDA including four of trastuzumab (Herceptin, Genentech), namely **Ogivri** (trastuzumab, dkst); **Herzuma** (trastuzumab, pkrb); **Ontruzant** (trastuzumab, dttb) and Trazimera (trastuzumab, qyyp) and two of rituximab, viz., Ruxience (rituximab, Pfizer) and Truxima (rituximab, Teva and Celltrion) [10, 11]. Also, more than 20 biosimilars have been approved by the European Medicines Agency, including those of monoclonal antibodies like infliximab, etanercept and adalimumab [12, 13]

Biosimilars of bevacizumab (Avastin: Roche Pharma AG) have also been studied extensively including bevacizumab-awwb (Mvasi, Amgen and Allergan) and bevacizumab-bvzr (Zirabev, Pfizer) and have already been approved by the FDA for treatment of recurrent GBM [14, 15]. In countries like India, where high cost and hence affordability is the prime issue with use of original molecule, biosimilars play a pivotal role in the disease management. The brands of bevacizumab currently available at our centre are—Avastin: Roche Pharma AG (original/innovator), BevaciRel: Reliance Life sciences and Bryxta: Zydus Oncosciences (biosimilar). The costs of 100 mg and 400 mg vials of Avastin are approximately 20,000 Indian rupees (260 $), 82,000 Indian rupees (1100 $), whereas of BevaciRel are 8,500 Indian rupees (110 $), 35,000 Indian rupees (460 $) and of Bryxta are 8,500 Indian rupees (110 $), 26,000 Indian rupees (340 $), respectively. Innovator bevacizumab was offered only to those patients who had either medical or employer insurance, who could afford on the basis of their income, social status and physical judgement. Non-affording patients were not given option of innovator bevacizumab. Hence, none of our patients receiving innovator changed to biosimilar bevacizumab and also no one has discontinued biosimilar bevacizumab due to non-affordability.

This huge difference in the cost gets reflected on the affordability and the same was also noted in our observational study. Here, we found a statistically significant difference between the two groups in terms of patients in the two categories (private and general), Fisher’s exact test (*p* value)—0.028. Forty-two out of total 57 patients (73.7%) in the innovator group belonged to the private category while for the biosimilar group, this figure was 13 out of 25 patients (52%) clearly indicating that mostly the private category patients could afford the innovator drug.

Monk *et al* [16] conducted a survey to identify the barriers to bevacizumab access in the US, European countries and emerging markets (EM: Brazil, Mexico and Turkey). Bevacizumab was indicated as a second line drug in the treatment of GBM in the US and EM. Most of the physicians from EM reasoned the lack of reimbursement and high costs for its limited use. Also nearly 50% of the physicians admitted that they would ‘definitely’ or ‘probably’ prescribe biosimilar of bevacizumab, if available [16].

This highlights the importance and need of introducing low cost biosimilars to address this problem area with intent to cause greatest impact on the patient outcomes.

IDH mutation, 1p/19q co-deletion, MGMT methylation and TERT promoter mutations are considered prognostic markers in diffuse glioma [17, 18]. In our study, there was significant difference between the innovator and biosimilar groups with regard to the above-mentioned mutations except IDH mutation; however, there was non-significant difference in OS and PFS between the two groups with HR of 1.21 (95% CI, 0.67 to 2.17; *p*-value = 0.51) and 0.61 (95% CI 0.35 to 1.05; *p*-value = 0.075), respectively. Likewise, Thatcher *et al* [19] in MAPLE study found similar PFS and OS with both ABP 215 (U.S.: MVASI (bevacizumab-awwb); European Union (EU):MVASI (bevacizumab)) and bevacizumab reference product in advanced non-small cell lung cancer patients, with estimated HR of 1.03 (90% CI, 0.83–1.29) for PFS [19]. Apsangikar *et al* [20] also found biosimilar bevacizumab (BevaciRel: Reliance Life sciences) to be non-inferior to the reference bevacizumab in metastatic colorectal cancer [20].

Additionally, these biosimilar drugs also have proved their worth in terms of safety and toxicities when compared to the original molecule. In the MAPLE study, the frequency, type and severity of adverse events were comparable between biosimilar and reference bevacizumab and were same as expected of the latter [19]. Apsangikar *et al* [20] also observed similar adverse events with both the biosimilar bevacizumab and the reference drug. [20]. Similarly, in our study, we observed the common anti-VEGF associated toxicities with both the drugs including similar grade 3 and 4 toxicities. Also, the difference in the safety profiles between the two groups was statistically non-significant. There was no death due to adverse events of the drug in either group.

First study to analyse the efficacy of bevacizumab biosimilars in recurrent or progressive glioblastoma patients makes it distinctive. However, retrospective nature of the study limits the study. In future, phase 3 randomised controlled trials can be planned to more appropriately explore this important research area.

## Conclusion

In brain tumour patients, both the innovator and biosimilar bevacizumab seem to have similar clinical efficacy and safety. Prospective studies in this direction may provide greater insight into the subject.

## List of abbreviations

TTP, Time to progression; OS, Overall survival; PFS, Progression free survival; ECOG-PS, Eastern Cooperative Oncology Group-Performance Status; VEGF, Vascular endothelial growth factor; FDA, Food and Drug Association; GCP, Good clinical practice; ICH, International Council for Harmonisation of Technical Requirements for Pharmaceuticals for Human Use. IDH, Isocitrate dehydrogenase; MGMT, O6-methylguanine-DNA methyl-transferase; GBM, Glioblastoma Multiforme.

## Declarations

### Ethics approval and consent to participate

The study methodology was approved by Institutional Ethics Committee-III, Advanced Centre for Treatment, Research and Education in Cancer (ACTREC), Mumbai-410210, India. Waiver of consent was obtained. Principle of declaration of GCP and International Council for Harmonisation of Technical Requirements for Pharmaceuticals for Human Use (ICH) was obtained. All patients were provided written informed consent prior to chemotherapy.

## Consent for publication

We are giving consent for publication.

## Availability of data and material

The authors confirm that the data supporting the findings of this study are available within the article.

## Conflicts of interests

The authors declare that they have no competing interests.

## Funding

None.

## Authors’ contributions

All authors contributed to the study conception and design. Material preparation, data collection and analysis were performed by all. The first draft of the manuscript was written by Vijay Maruti Patil and all authors commented on previous versions of the manuscript. All authors read and approved the final manuscript.

## Figures and Tables

**Table 1.** Baseline characteristics.

Characteristics	Innovator	Biosimilar	Fisher’s exact test (*p*-value)
Non-elderly (up to 59 years) Elderly (≥60 years)	50 (87.7%) 7 (12.3%)	20 (80%) 5 (20%)	0.498
ECOG-PS 1 2 3 4 Not recorded	21 (38.8%) 7 (12.3%) 13 (22.8%) 4 (7%) 12 (21.1%)	8 (32%) 8 (32%) 6 (24%) 1 (4%) 2 (8%)	0.241
GenderMaleFemale	44 (77.2%) 13 (22.8%)	20 (80%) 5 (20%)	0.791
CategoryPrivateGeneral	42 (73.7%) 15 (26.3%)	13 (52%) 12 (48%)	0.028
HypertensionNo Yes	47 (82.5%) 10 (17.5)	23 (92%) 2 (8%)	0.328
DiabetesNo Yes	50 (87.7%) 7 (12.3%)	22 (88%) 3 (12%)	1.0
IHD NoYes	56 (98.2%) 1 (1.8%)	25 (100%) 0 (0%)	1.0
Smoker NoYes	57 (100%) 0(0%)	24 (96%) 1 (4%)	0.305

ECOG-PS, Eastern Cooperative Oncology Group-Performance Status IHD, Ischemic heart disease

**Table 2.** Molecular markers comparison between biosimilar and innovator bevacizumab.

	Absent	Present	Not done	Equivocal	Un-interpretable
**IDH mutation (*p*-value = 0.143)**					
Biosimilar	14 (56%)	7 (28%)	3 (12%)	1 (4%)	0
Innovator	21 (36.8%)	16 (28.1%)	19 (33.3%)	1 (1.8)	0
**MGMT methylation (*p*-value = 0.007)**					
Biosimilar	15 (60%)	7 (28%)	3 (12%)	0	0
Innovator	18 (31.6%)	10 (17.5%)	26 45.6%)	3 (5.3%)	0
**1p19q deletion (*p*-value = 0.001)**					
Biosimilar	15 (60%)	0	10 (40%)	0	0
Innovator	11 (19.3%)	2 (3.5%)	44 (77.2%)	0	0
**TERT promoter mutation (*p*-value = 0.000)**					
Biosimilar	15 (60%)	0	10 (40%)	0	0
Innovator	4 (7%)	0	53 (93%)	0	0

**Table 3.** Toxicities comparison between biosimilar and innovator bevacizumab.

Toxicities	Grade 1	Grade 2	Grade 3	Grade 4	Grade 5
**Febrile neutropenia**					
Biosimilar	25 (100%)	0	0	0	0
Innovator	54 (94.7%)	0	2 (3.55)	1 (1.8%)	0
**Diarrhoea**					
Biosimilar	24 (96%)	1 (4%)	0	0	0
Innovator	50 (87.7%)	1 (1.8%)	5 (8.8%)	1 (1.8%)	0
**Myelosuppression**					
Biosimilar	25 (100%)	0	0	0	0
Innovator	51 (91.2%)	2 (3.5%)	2 (3.5%)	1 (1.8%)	0
**Transaminitis**					
Biosimilar	23 (92%)	2 (8%)	0	0	0
Innovator	49 (85.9%)	4 (7%)	3 (5.3%)	1 (1.8%)	0
**Dyslipidaemia**					
Biosimilar	24 (96%)	1 (4%)	0	0	0
Innovator	48 (84.2%)	7 (12.3%)	2 (3.5%)	0	0
**Bleeding**					
Biosimilar	25 (100%)	0	0	0	0
Innovator	51 (89.5%)	4 (7%)	2 (3.5%)	0	0
**Thrombosis**					
Biosimilar	25 (100%)	0	0	0	0
Innovator	54 (94.7%)	1 (1.8%)	2 (3.5%)	0	0
**Proteinuria**					
Biosimilar	25 (100%)	0	0	0	0
Innovator	53 (93%)	2 (3.5%)	2 (3.5%)	0	0
**Hypersensitivity**					
Biosimilar	25 (100%)	0	0	0	0
Innovator	54 (94.7%)	1 (1.8%)	2 (3.5%)	0	0
**Hypertension**					
Biosimilar	25 (100%)	0	0	0	0
Innovator	49 (86%)	3 (5.3%)	5 (8.8%)	0	0
**Fatigue**					
Biosimilar	25 (100%)	0	0	0	0
Innovator	54 (94.7%)	0	2 (3.5%)	1 (1.8%)	0
